# In Vitro and In Vivo Evaluation of ^99m^Tc-Polymyxin B for Specific Targeting of Gram-Bacteria

**DOI:** 10.3390/biom11020232

**Published:** 2021-02-05

**Authors:** Sveva Auletta, Filippo Galli, Michela Varani, Giuseppe Campagna, Martina Conserva, Daniela Martinelli, Iolanda Santino, Alberto Signore

**Affiliations:** 1 Nuclear Medicine Unit, Department of Medical-Surgical Sciences and of Translational Medicine, “Sapienza” University of Rome, 00161 Rome, Italy; sveva.auletta@hotmail.it (S.A.); filippo.galli@hotmail.com (F.G.); varanimichela@gmail.com (M.V.); gius.campagna@gmail.com (G.C.); martina.conserva977@gmail.com (M.C.); 2 Microbiology Unit, Department of Clinical and Molecular Medicine, “Sapienza” University of Rome, 00161 Rome, Italy; dany.stefano@tiscali.it (D.M.); iolanda.santino@uniroma1.it (I.S.)

**Keywords:** polymyxin B, infection imaging, bacteria, ^99m^Tc-polymyxin B

## Abstract

Background: Infectious diseases are one of the main causes of morbidity and mortality worldwide. Nuclear molecular imaging would be of great help to non-invasively discriminate between septic and sterile inflammation through available radiopharmaceuticals, as none is currently available for clinical practice. Here, we describe the radiolabeling procedure and in vitro and in vivo studies of ^99m^Tc-polymyxin B sulfate (PMB) as a new single photon emission imaging agent for the characterization of infections due to Gram-negative bacteria. Results: Labeling efficiency was 97 ± 2% with an average molar activity of 29.5 ± 0.6 MBq/nmol. The product was highly stable in saline and serum up to 6 h. In vitro binding assay showed significant displaceable binding to Gram-negative bacteria but not to Gram-positive controls. In mice, ^99m^Tc-HYNIC-PMB was mainly taken up by liver and kidneys. Targeting studies confirmed the specificity of ^99m^Tc-HYNIC-PMB obtained in vitro, showing significantly higher T/B ratios for Gram-negative bacteria than Gram-positive controls. Conclusions: In vitro and in vivo results suggest that ^99m^Tc-HYNIC-PMB has a potential for in vivo identification of Gram-negative bacteria in patients with infections of unknown etiology. However, further investigations are needed to deeply understand the mechanism of action and behavior of ^99m^Tc-HYNIC-PMB in other animal models and in humans.

## 1. Introduction

Discrimination between sterile inflammation and infection has always been one of the major challenges for the scientific community, and for nuclear medicine too. Several radiopharmaceuticals, such as antimicrobial peptides, antibiotics, sugars or antifungal, do not allow differentiating between infection and sterile inflammation or unmasking sites of occult infection. Despite excellent pre-clinical results, none of these radiopharmaceuticals has been introduced into clinics yet, because of poor specificity in humans [1,2,3,4,5].

In fact, radiolabeled leukocyte imaging, with either ^99m^Tc-HMPAO or ^111^In-oxine, is the scintigraphic imaging test of choice for most infections in the immunocompetent population [6,7].

In some cases, such as in spondylodiscitis, the use of [^18^F] FDG has proved to be more sensitive and specific than radiolabeled white blood cells [8]. Nevertheless, when infection is diagnosed, the problem remains about the identification of the causative agent, and hemocultures or needle aspiration (or biopsy) are often necessary to isolate the pathogen. However, ultrasound- or CT-guided biopsies or fluid aspiration can result in higher specificity but always with low sensitivity, ranging from 69% to 80% [9,10,11,12,13].

**Figure 1:**
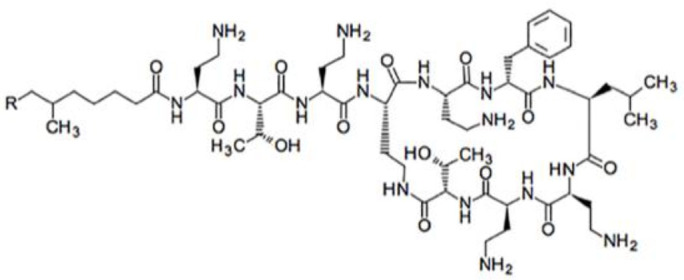
Structural formula of native PMB with a molecular weight of approximately 1200 g/mole.

A major improvement for therapy would be to identify, by a simple imaging modality, if the infection is caused by Gram-negative (Gram-) or Gram-positive (Gram+) bacteria, or if it is a fungal infection. In the last five years, we aimed at synthetizing a new radiopharmaceutical for the specific identification in vivo, by gamma camera imaging, of Gram- infections. Among the many antimicrobial peptides, we concentrated on polymyxins [14,15,16]. Polymyxins (A, B, C, D, E or colistin) are decapeptides with molecular weights in the range of 1200 Da that differ only for few amino acid residues [17]. This class of antimicrobial peptides is characterized from a specific structure consisting of a cyclic heptapeptide ring bound, through a tripeptide side chain, to a hydrophobic fatty acid tail (Figure 1). Even though five polymyxins have been described, only polymyxin B and colistin are used for clinical purpose [18].

Studies conducted on the structure–activity relationship of polymyxin B (PMB), demonstrate that PMB acts on lipopolysaccharide (LPS) as an amphipathic antimicrobial peptide: the polar face of the peptide interacts with the polar lipid A component of LPS, while the lipophilic face permeates into the hydrophobic layer of the outer membrane, resulting in disruption of the membrane and in a major susceptibility to other hydrophobic antibiotics [19,20].

Commercially, polymyxin B is available as polymyxin B sulfate, a mixture of polymyxin B_1_ and B_2_ as prevalent forms and polymyxins B_3–6_, which differ only for the fatty acid tail [21].

In the last decades, the use of polymyxin B and colistin was renewed due to increase of multidrug-resistant (MDR) Gram- bacterial infections [22] such as *Pseudomonas aeruginosa* and *Acinetobacter baumanii,* which are resistant to many available antibiotics [23,24].

The interaction between antimicrobial peptides, such as PMB, and microbial plasmatic membrane is initially driven by electrostatic bounds between anionic and cationic charges, on lipid bacterial leaflet and peptide, respectively [25]. Then, the amphipathic action of antimicrobial peptide induces hydrophobic interactions with consequent formation of pores that lead to internalization of antimicrobial peptide followed by binding to intracellular molecules such as LPS of dead bacteria.

Because of the considerable potential of this antimicrobial peptide, in this paper, we describe the radiolabeling of polymyxin B sulfate with 99m-Technetium (^99m^Tc) with the aim to produce a new radiopharmaceutical, with high specific activity, for imaging of Gram- infections. This would allow performing in vivo studies, avoiding pharmacological effects.

## 2. Materials and Methods

### 2.1. Conjugation

Labeling of polymyxin B sulfate (Sigma Aldrich, St. Louis, MO, USA) was performed with indirect method: PMB molecules were conjugated with a heterobifunctional crosslinker, succinimidyl-6-hydrazinonicotinate hydrochloride (HYNIC), purchased from ABX (advanced biochemical compounds, Radeberg, Germany). HYNIC is able to react with free ε-amino groups of lysine in proteins and to chelate ^99m^Tc [26].

HYNIC was dissolved in dimethylformamide (70 µM) (DMF; Sigma-Aldrich, St. Louis, MO, USA) and PMB was dissolved in water. They were incubated for 2 h in the dark at room temperature using different HYNIC:protein molar ratios. To eliminate free SH-NH molecules, the reaction mixture was purified by PD MidiTrap G-10 (GE Healthcare, Waukesha, WI, USA) using distilled water as eluent. The amount of PMB in each fraction was initially determined by bicinchoninic acid (BCA) assay: 25 µL of purified samples were added to 200 µL of BCA reagents in a microplate and incubated at 37 °C for 30 min. Subsequently, absorbance at 562 nm was measured with a microplate spectrophotometer (Thermo Fisher Scientific Inc, Waltham, MA, USA) and compared with protein solutions of known concentration.

In other experiments, the amount of PMB in fractions was measured by reverse phase HPLC chromatography by standardizing an automatic method for precise quantification of eluted protein on the basis of absorbance at 210 nm, as described below. When compared, BCA assay and HPLC gave identical results. The conjugated product was also analyzed by mass spectrometry (MALDI-TOF).

### 2.2. Radiolabeling Procedure

Briefly, 10 µg of conjugated PMB (50 µL) were labeled with 222 MBq of freshly eluted ^99m^TcO_4_ (100 µL NaCl 0.9%). The reaction was conducted in the presence of different amounts of co-ligand tricine (in 75 µL) and reducing agent stannous chloride (SnCl_2_) (in 25 µL), in order to obtain the best labeling conditions. Tricine (Sigma-Aldrich, St. Louis, MO) was dissolved in distilled water and SnCl_2_ (Sigma-Aldrich, St. Louis, MO, USA) in purged HCl 0.1 M (10 mg/mL). The final labeling volume was 250 µL.

The reaction solution was incubated for 10 min at room temperature and the labeling efficiency (LE) and colloid percentages were evaluated by quality controls.

### 2.3. Quality Controls

LE and colloids percentage were evaluated by instant thin layer chromatography (ITLC) and high-performance liquid chromatography (HPLC).

For iTLC, silica gel strips (Pall LifeSciences, Port Washington, NY, USA) were used as stationary phase, NaCl 0.9% solution as mobile phase for determination of free pertechnetate (Rf = 0.9) and NH_3_:H_2_O:EtOH (1:5:3) solution as mobile phase for colloids (Rf = 0.1) determination. iTLC strips were analyzed by a linear radio-scanner equipped with a collimated gamma-ray detector (Bioscan Inc, Poway, CA, USA) and each species was determined. Scan time for a 10 cm strip was 2 min.

HPLC was performed with a Gilson system, using a reverse phase chromatography C-18 column (5 mm, 5 µm, 250 × 4.6 mm, Phenomenex, Torrance, CA, USA) and a H_2_O (A)/Acetonitrile (B) (Baker, Sanford, ME, USA) gradient (0–5 min 5% B; 5–15 min 5–95% B; 15–18 min 95% B; 18–21 min 95–5% B) with a flow rate of 1 mL/min.

Stability assay was performed adding 100 µL of ^99m^Tc-HYNIC-PMB to 900 µL of freshly prepared human blood serum or NaCl 0.9%. The vials were incubated at 37 °C, and the radiochemical purity was measured at 1, 3, 6 and 24 h by HPLC.

### 2.4. Micro-Organisms

The laboratory strains *Escherichia coli* (ATCC 25922), *P. aeruginosa* (ATCC 27853), *Staphilococcus aureus* (ATCC 25923), *Enterococcus faecalis* (ATCC 29212), *A. baumanii* (ATCC 19606) and *Klebsiella pneumoniae* (ATCC 13883) were used. Bacteria were stored at −70 °C using a cryovial bead preservation system. Single cryovial beads were cultured overnight on Brain Heart Infusion Agar (BHI) for 24 h and then cultured on blood agar plates to evaluate the replication rate. For in vitro studies, a known concentration of bacteria was incubated until reaching the desired concentration of 1 × 10^8^ CFU.

### 2.5. In Vitro Binding Studies

Binding of ^99m^Tc-HYNIC-PMB to all bacterial strains were tested in vitro. ^99m^Tc-HYNIC-PMB was diluted 1:100 in NaCl 0.9% and 250 μL were transferred to vials pre-filled with 500 μL of bacteria (10^8^ CFU) and the correct volume of NaCl 0.9% + 1% of bovine serum albumin (BSA) to reach a final volume of 1 mL. Vials with bacterial cells were incubated at 37 °C and 4 °C to study whether the temperature influences the binding. Binding assay was also performed in the presence and in the absence of 100-fold excess of unlabeled PMB to investigate the displacement of the radiopharmaceutical. The binding to bacteria was calculated at different time points (10 min, 30 min and 1 h) by centrifugation of vials for 10 min at 20,000× *g* at 4 °C. Pellets were washed with 1 mL of NaCl 0.9% + 1% of BSA and centrifuged again for 10 min at 20,000× *g*. Pellets were then re-suspended in 1 mL of NaCl 0.9% + 1% of BSA. Supernatants and re-suspended pellets were counted in a single-well NaI γ-counter (AtomLab, 500-Biodex) and the counts per minute (CPM) recorded. The percentage of ^99m^Tc-HYNIC-PMB in the pellets was calculated as CPM/CPM_0_, where CPM were associated to pellets and CPM_0_ the CPM of pellet plus CPM of supernatant.

### 2.6. Biodistribution Studies

All applicable institutional and/or national guidelines for the care and use of animals were followed.

The physiological distribution of ^99m^Tc-HYNIC-PMB was determined in healthy C57BL/6 mice (female, 6–10 weeks old, Envigo). Approximately 1.85 MBq (50 µL, 0.1 mg) of radiolabeled PMB was injected in the lateral tail vein of mice. The exact injected activity was calculated by dilution of the original sample and weighing the syringe before and after sampling and after injection. All syringes used were without dead volume (BD Micro-FineTM+). Images were acquired under anesthesia using a high resolution γ-camera (Li-Tech, Italy) [27] for 25 s at 1, for 31 s at 3 h and for 45 s at 6 h.

After each time point, four mice were sacrificed; blood samples and major organs (small bowel, large bowel, kidneys, spleen, stomach, liver, muscle, bone, lungs and salivary glands) were collected and weighted for ex-vivo studies. The radioactivity in each vial was counted in a single-well gamma counter (2470 Wizard Automatic Gamma Counter, PerkinElmer, Waltham, MA, USA).

Radioactivity in all organs was expressed as percentage of injected dose per organ (%ID) and percentage of injected dose per gram (%ID/g).

### 2.7. Targeting Studies

The specificity of ^99m^Tc-HYNIC-PMB to localize infectious foci was investigated in C57/BL6 mice (female, 6–10 weeks old, Envigo). The infection was inducted by the injectable 107. 10^8^ and 10^9^ CFU for *E. coli*, *P. aeruginosa*, *A. baumanii*, *S. aureus* and *E. faecalis*) in right thigh in 100 µL of extracellular matrix (ECM)-based hydrogel (Matrigel^®^, Corning, New York, NY, USA). This compound allows obtaining a focused and high concentration infection in the mouse thigh. As control, mice received an injection of ECM-based hydrogel alone in the contralateral thigh with the aim to induce a sterile inflammatory reaction, as previously demonstrated [28]. For each dose of bacteria, four mice were used to have reproducible and statistically significant data. Imaging was performed 24 h after the injection of bacteria at 1, 3 and 6 h after the injection of ^99m^Tc-HYNIC-PMB in the lateral tail vein (1.85 MBq, 50 µL, 0.1 µg), as described in the previous section. Planar images were acquired under anesthesia. After imaging session at 6 h, mice were sacrificed. From each infected thigh, we removed the infected area that resulted inflamed at visual inspection. From contralateral thigh, we removed an equivalent volume of tissue where ECM-based hydrogel was administered. All removed tissues were weighed and counted using a single-well gamma counter (2470 Wizard Automatic Gamma Counter, PerkinElmer, Waltham, MA, USA).

A few mice were also studied up to 24 h p.i., but the best time points for all experiments were set at 3 and 6 h p.i. due to rapid binding of PMB to bacteria.

The radioactivity was expressed as percentage of injected dose per organ (%ID) and percentage of injected dose per gram (%ID/g). For each time point, the in vivo target-to-background ratios (T/B ratios) were measured by calculating the activity in an irregular region of interest (ROI) over the infected thigh (target) and in a mirrored ROI of same shape and size, for the contralateral non-infected thigh (background).

### 2.8. Statistical Analysis

Statistical analysis was performed using SAS v. 9.4 (SAS, Institute Inc., Cary, NC, USA). All results are shown as mean ± SD. Shapiro–Wilk test was used to verify the normality of distribution of continuous variables. Comparisons of in vitro binding results were analyzed by Student t-test (HOT vs. 100× cold). Multiple comparisons were performed by Benjamini–Hochberg (FDR). A probability level of *p* < 0.05 was considered statistically significant.

## 3. Results

### 3.1. Radiolabeling

**Figure 2:**
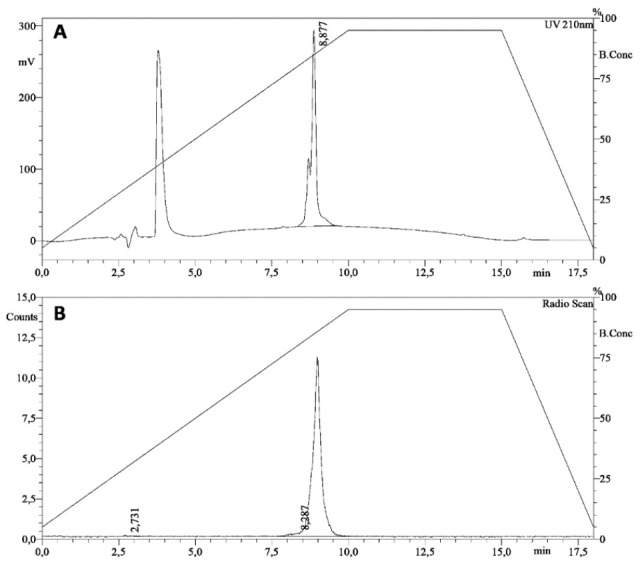
HPLC chromatogram of ^99m^Tc-HYNIC-PMB: (**A**) UV chromatogram; and (**B**) radioactive chromatogram.

**Figure 3:**
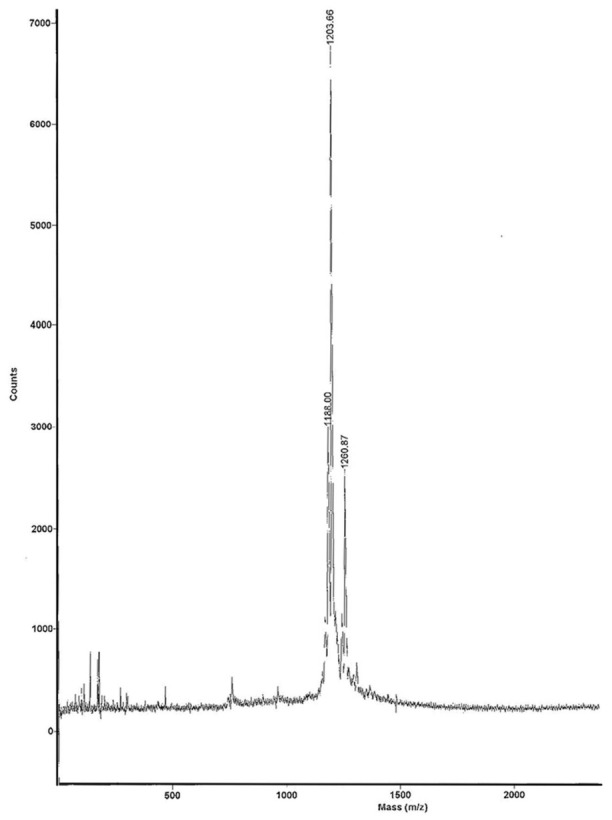
Mass spectrometry analysis of conjugation HYNIC-PMB. The graph shows one peak corresponding to unconjugated PMB at 1203.6 *m*/*z* and one peak of HYNIC-conjugated PMB at 1260.8 *m*/*z*.

The highest labeling efficiency (LE) was obtained using HYNIC:PMB molar ratio of 1.5:1, tricine:SnCl_2_ molar ratio of 50:1, obtaining a LE of 97 ± 2% and an amount of colloids <10%, as shown in Figure 2 and Figure 3.

The molar activity is equal to 29.5 ± 0.6 MBq/nmol (21.7 ± 0.4 MBq/µg). Radiolabeled PMB was stable up to 6 h both in human serum and in a 0.9% NaCl solution at 37 °C (Table 1).

**Figure 4:**
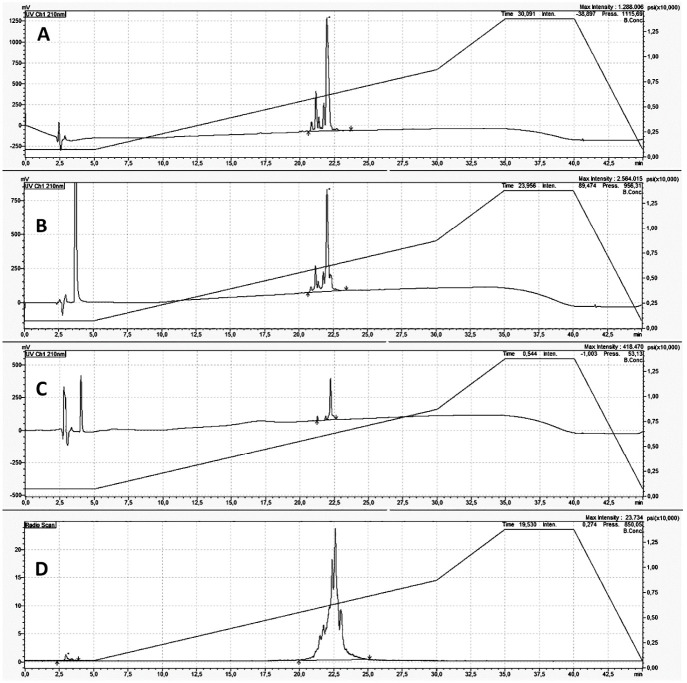
HPLC analysis of different forms of PMB. Acetonitrile gradient over 45 min. Different forms of PMB: chromatogram (UV 210 nm) of unlabeled PMB (**A**); chromatogram (UV 210 nm) of HYNIC-conjugated PMB (**B**); chromatogram (UV 210 nm) of ^99m^Tc-HYNIC-PMB (**C**); and Radiogram (counts) of ^99m^Tc-HYNIC-PMB (**D**). The elution profiles of unlabeled and labeled PMB were unmodified using a faster Acetonitrile gradient (21 min), as shown in Figure 2.

MALDI-TOF analysis showed one peak corresponding to unconjugated PMB at ratio mass-to-charge (*m*/*z*) equal to 1203.66 and one more peak of HYNIC-conjugated PMB at 1260.87 *m*/*z* (Figure 4). These data demonstrate that only one molecule of HYNIC is conjugated to PMB and presumably at the same position, as also confirmed by HPLC analysis showing only one peak of conjugated and radiolabeled PMB.

### 3.2. In Vitro Binding Studies

The binding test of ^99m^Tc-HYNIC-PMB to different bacterial strains is shown in Table 2.

Regarding the binding to *P. aeruginosa, S. aureus and E. faecalis*, the results show that the temperature does not influence the binding. Instead, the binding to *E. coli* is influenced by temperature, as well as slightly for *A. baumanii* and *K. pneumoniae*. Specific displaceable binding was observed in Gram- bacteria (*E. coli*, *P. aeruginosa* and *A. baumanii*) at 37 °C and 4 °C (between 56% and 86% displaceable). In Gram+ bacteria (*K. pneumoniae*, *S. aureus* and *E. faecalis*), binding was generally lower and poorly displaceable (between 12% and 47%).

### 3.3. Biodistribution Studies

Biodistribution studies exhibit high uptake by the kidneys and lower signal from liver and spleen (Table 3).

Single organ counting showed an accumulation at renal level and a large bowel activity increasing over time.

**Figure 5:**
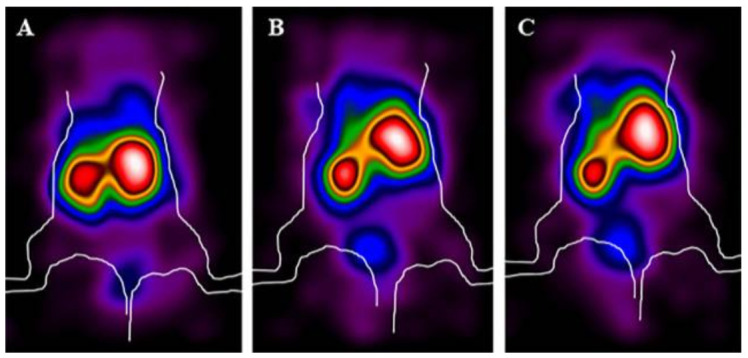
Representative planar γ-camera images of whole-body of healthy mice: 1 h (**A**); 3 h (**B**); and 6 h (**C**) p.i. of ^99m^Tc-HYNIC-PMB showing uptake mainly in liver and kidneys with minimal excretion in the urine (bladder) over time.

Figure 5 shows the increase of activity over time in the bladder, indicating that renal excretion also occurs.

### 3.4. Targeting Studies

**Figure 6:**
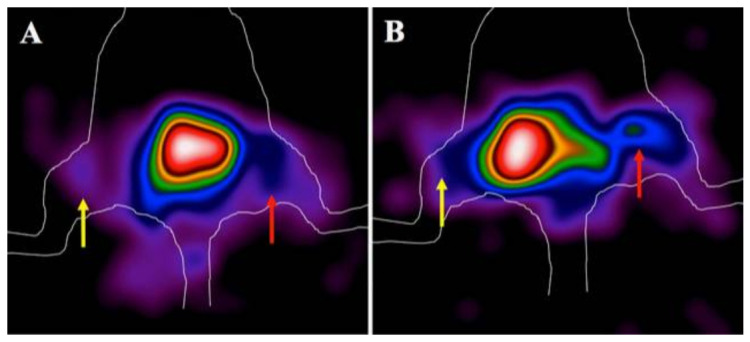
Representative planar γ-camera images of zoom of lower body part of two mice infected with 10^9^ CFU of S. aureus (**A**) (red arrows) and P. aeruginosa (**B**) (red arrows) versus contralateral thigh with only (ECM)-based hydrogel (yellow arrows) at 6 h p.i. of ^99m^Tc-HYNIC-PMB.

Figure 6 shows a representative image of uptake in the infectious focus in comparison to contralateral by zooming on lower body part of mice, acquired by high resolution planar γ-camera. In particular, *S. aureus* (Figure 6A) and *P. aeruginosa* (Figure 6B) were chosen as representative images of uptake at 6 h p.i. and using 10^9^ CFU. It is possible to appreciate how ^99m^Tc-HYNIC-PMB accumulates more in the P. aeruginosa lesion (right thigh) than in the contralateral left thigh or in the S. aureus infected mouse.

From these images, T/B ratios were measured from the image pixel matrix for each mouse of each experiment, which showed a slight increase over time for Gram- bacteria (*E. coli*, *P. aeruginosa* and *A. baumanii*) and a flat trend for Gram+ bacteria (*S. aureus* and *E. faecalis*).

**Figure 7:**
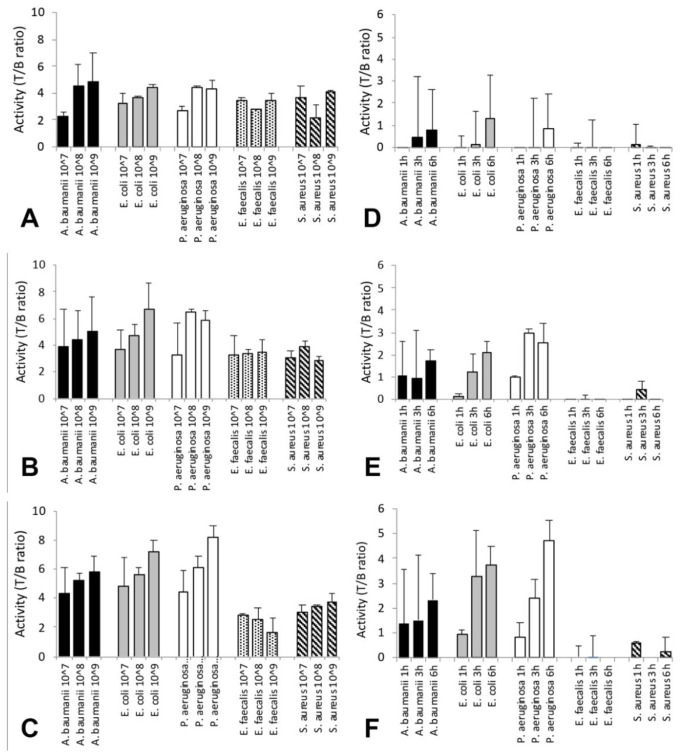
In vivo T/B ratios of ^99m^Tc-HYNIC-PMB at different time points with different amounts of bacteria (10^7^, 10^8^, and 10^9^). Time points: 1 h (**A**,**D**); 3 h (**B**,**E**); and 6 h (**C**,**F**) p.i. of *A. baumanii* (black), *E. coli* (grey), *P. aeruginosa* (white), *E. faecalis* (black dots), and *S. aureus* (black stripes). In (**A**–**C**), values are mean ± SD of calculated T/B ratios; multiple comparison of Gram-negative (*A. baumanii*, *E. coli* and *P. aeruginosa*) vs. Gram-positive (*E. faecalis* and *S. aureus*) was performed, as reported in Table 4. In (**D**–**F**) the same values as in (**A**–**C**), but after subtraction of background activity (−3.5) to emphasize different uptake per time point and bacteria. Only 10^8^ and 10^9^ Gram- bacteria can be seen at 1, 3 and 6 h p.i. By contrast, 10^7^ bacteria can be detected only at 6 h p.i. Statistical analysis is summarized in Table 4.

At all time points, using different CFU of Gram- bacteria, an increase of T/B ratios in relation to CFU can be seen. Conversely, for Gram+ bacteria, the increasing trend was not observed in relation to the increasing number of bacteria (Figure 7).

Indeed, there are statistically significant differences between T/B ratios of Gram- strains when compared to Gram+, especially at 6 h p.i., as reported in Table 4.

The results of ex-vivo counting of infected and contralateral thighs did not show the same results as obtained in vivo (data not shown) because of difficulty in identifying the infected area to remove.

For this reason, these data were considered non-reliable and only in vivo calculated T/B are shown in Figure 7.

## 4. Discussion

In the last decade, many studies have been published about new radiopharmaceuticals able to localize infective foci by direct interaction with bacterial cells, including antimicrobial peptides, antibiotics, phages, immunoglobulins or sugars, but none of these showed high specificity or sensibility.

Many radiolabeled antibiotics have been proposed in humans, but none can really be considered “infection-specific” because of low specificity, low selectivity for a precise bacterial strain and lack of specific binding to bacteria [29]. Antimicrobial peptides, mostly UBI (29–41), have been intensively studied for bacterial infection imaging, first radiolabeled with ^99m^Tc for SPECT and then with ^68^Ga for positron emission tomography (PET). Nevertheless, no conclusive results have been produced due to differences in infection models, bacterial strains and imaging protocol in preclinical and clinical studies [30,31,32,33,34,35,36,37,38,39].

All these approaches aimed at finding a new, easy to use, radiopharmaceuticals for imaging infections (due to both Gram+ and Gram- bacteria) as an alternative to well-established scintigraphy with radiolabeled white blood cells (WBC) that involves patient’s blood separation and several acquisitions over time. Nevertheless, the diagnostic accuracy of labeled WBC is between 90% and 98% for differential diagnosis between infection and sterile inflammation, and there is no need for developing a new radiopharmaceutical for infection imaging [8].

Our approach is different. We did not aim at developing an alternative to labeled WBC, but we aimed to obtain a second-line test to discriminate between Gram- and Gram+ infections to provide substantial help to clinicians for starting an appropriate antibiotic therapy, in the case the pathogen cannot be isolated. The same strategy was followed by Weinstein and colleagues who used ^18^F-fluorodeoxysorbitol ([^18^F]FDS) to selectively image Gram- bacteria [40]. However, due to the different isotope and different animal model used, we cannot compare our results with those obtained by Weinstein et al. [40].

After extensive searching and several attempts, we selected PMB [41] to develop a new radiopharmaceutical for the non-invasive diagnosis of selective Gram-negative infection by gamma camera imaging. Herein, PMB has been conjugated with HYNIC as bifunctional crosslinker and radiolabeled with ^99m^Tc, by using tricine as co-ligand, although others (e.g., EDDA) were also considered at an early stage. These radiolabeling conditions led to a radiopharmaceutical with high specific activity, labeling efficiency, stability and specificity for Gram- bacteria, as demonstrated by in vitro binding studies on several bacterial strains (*P. aeruginosa*, *A. baumanii* and *K. pneumonia* as Gram- and *S. aureus* and *E. faecalis* as Gram+).

Based on in vitro results, we performed in vivo biodistribution studies in healthy mice that showed multiple excretion routes, as also suggested by another study [42]. Indeed, ^99m^Tc-HYNIC-PMB metabolism could be mainly hepatic (as suggested by the increasing fecal activity in the large bowel, over time), whereas the apparently stable renal activity over time could be due to a non-specific renal uptake mechanism, although some renal excretion can also occur (as suggested by the increasing bladder activity, over time). This activity in the bladder does not correspond to an increase of activity in the stomach and salivary glands, which, on the contrary, is considerably reduced over time (Table 3). These findings suggest that some renal excretion of ^99m^Tc-HYNIC-PMB, or of a ^99m^Tc-labeled degradation product, occurs.

Furthermore, we performed in vivo targeting studies, inducing infection with the same Gram- and Gram+ bacterial strains, injected with (ECM)-based hydrogel in the right thigh of the mouse. These experiments showed significantly higher uptake in the infectious site when using Gram- bacteria than Gram+ ones, in relation to both the increased number of bacteria and over time, as shown by T/B ratios in Figure 6. In particular, the best time point for imaging mice was 6 h p.i. and the best number of CFU detected was 10^8^ to 10^9^. Nevertheless, Gram+ bacteria also showed a non-specific uptake, which should be considered in the case of human studies as potential factor that may reduce the sensitivity of imaging. If non-specific binding is subtracted from the data in Figure 4, it appears more evident that 10^8^ and 10^9^ Gram- bacteria can be visualized at 1, 3 and 6 h p.i., but 10^7^ bacteria can only be detected at 6 h p.i. (Figure 7D–F). In these graphs, the background threshold is randomly selected, but, mostly importantly, the graphs show that a Gram- infection can be distinguished from a Gram+ infection by considering an uptake over a certain threshold. The level of the threshold will probably depend on the type of infection, the site and animal model used.

The reason ex-vivo results of infected and contralateral thighs did not confirm the results obtained in vivo might be because the inflamed tissues to be removed from infected thighs were very difficult to identify and of variable size and weight. This can be due to a different degree of leukocytic infiltration and edema or generally to a host response to injury. Inflamed areas were generally larger in thighs infected with 10^9^ bacteria and in thighs infected with *A. baumanii* and smaller in Gram+ infected thighs. This variability in resected tissues resulted in a high variability of weights, and thus a high variability of %ID/g and T/B ratios. For this reason, these data were considered non-reliable in contrast to in vivo measurement of thigh activity by drawing the same ROI over the infected and non-infected thighs.

The best images were obtained using 10^8^ CFU of *P. aeruginosa*, at 3 and 6 h p.i., whereas, for *A. baumanii* and *E. coli*, more bacteria and later imaging time point (6 h p.i.) were needed to reach comparable results. Therefore, a detectability limit could exist for which bacterial amounts lower than 10^8^ CFU are more difficult to detect. We should also consider that, in humans, bacteria are spread (as in infected prosthesis and osteomyelitis) and not always localized (as in the case of endocarditis) and may also produce biofilm that may further reduce the sensitivity of the technique.

Therefore, a radiopharmaceutical with high specific activity is necessary to inject a reasonable amount of radioactivity, avoiding pharmacological side effects.

A possible criticism to the present study could be that the animal model we used does not well represent a human infection. However, we chose this model as an initial easy screening model for the evaluation of the specificity of ^99m^Tc-HYNIC-PMB. In the future, we will study a model of osteomyelitis [43] or a model with infected subcutaneous Teflon cage, as previously described [28] and according to recently published suggestions [44].

In addition, following the experience of ^68^Ga-radiolabeled antimicrobial peptides [5,45,46,47] and considering the fast binding of PMB to bacteria and fast metabolic clearance, we may consider labeling PMB with ^68^Ga for PET applications.

## 5. Conclusions

In the present study, we radiolabeled PMB with high specific activity, efficiency and stability. In vitro, the radiopharmaceutical showed a good specificity for Gram- bacteria in comparison to Gram+ ones as negative controls. In vivo, ^99m^Tc-HYNIC-PMB was excreted through multiple metabolic routes. Targeting studies confirmed the results obtained in vitro, showing statistically significant differences between Gram- and Gram+ infected mice and suggesting ^99m^Tc-HYNIC-PMB as potential agent for identification of Gram- infections. Further investigations are needed to investigate the in vivo sensitivity and specificity of ^99m^Tc-HYNIC-PMB in other animal models and in humans.

## Figures and Tables

**Table 1:** Stability of ^99m^Tc-HYNIC-PMB in NaCl and human serum.

0.9% NaCl			Human Serum		
1 h	3 h	6 h	1 h	3 h	6 h
99 ± 1.3%	99 ± 1.5%	98 ± 1.8%	97 ± 1.6%	96 ± 1.8%	96 ± 2.1%

**Table 2:** In vitro binding of ^99m^Tc-HYNIC-PMB in bacterial strains.

Bacterial Strain	37 °C		4 °C	
	HOT	+100× cold	HOT	+100× cold
*E. coli*	36.2 ± 12.5	9.9 ± 1.7	24.2 ± 8.6	10.4 ± 2.7
*P. aeruginosa*	31.5 ± 7.6 *	12.5 ± 5.3	32.7 ± 11.6	17.7 ± 9.8
*A. baumanii*	37.4 ± 0.9 **	5.4 ± 8.3	28 ± 4.3 **	7.3 ± 8.9
*K. pneumoniae*	45 ± 5.7 ***	20.6 ± 6.4	23.8 ± 2.3	20.2 ± 4.7
*S. aureus*	15.9 ± 9.2	12.6 ± 1.9	15.4 ± 7.8	12.6 ± 4.3
*E. faecalis*	18.5 ± 8.3	19.5 ± 4	14.8 ± 7.9	13 ± 1.3

Data are the percent CPM/CPM_0_ (mean ± SD) after 1 h incubation of ^99m^Tc-HYNIC-PMB with the different bacterial strains. Gram- bacteria (*E. coli*, *P. aeruginosa* and *A. baumanii*) and Gram+ bacteria (*K. pneumoniae*, *S. aureus* and *E. faecalis*). HOT, when only radiopharmaceutical was added to bacteria; 100× cold, when 100-fold molar excess of unlabeled PMB was added to bacteria together with tracer amount of radiopharmaceutical. Student *t*-test (HOT vs. 100× cold) for each experimental group (37 °C and 4 °C) = * *p* < 0.029; ** *p* < 0.01; *** *p* < 0.005.

**Table 3:** %ID/organ (mean ± SD) and %ID/g (mean ± SD) in tissues after ^99m^Tc-HYNIC-PMB injection, are shown in the upper and lower part of the table, respectively.

Organ	1 h	3 h	6 h
Blood	3.34 ± 0.51	1.87 ± 0.19	1.57 ± 0.18
Small Bowel	2.88 ± 0.21	2.01 ± 0.36	1.20 ± 0.01
Large Bowel	1.22 ± 0.22	1.62 ± 0.81	2.09 ± 0.41
Kidneys	163.92 ± 11.63	163.96 ± 31.41	154.42 ± 38.78
Spleen	11.95 ± 2.27	8.52 ± 8.28	12.95 ± 4.26
Stomach	1.87 ± 0.15	1.05 ± 0.30	0.65 ± 0.28
Liver	14.50 ± 0.64	10.89 ± 4.84	12.67 ± 0.71
Muscle	1.48 ± 0.45	0.63 ± 0.13	0.67 ± 0.07
Bone	3.02 ± 0.30	1.55 ± 0.41	1.38 ± 0.32
Lungs	5.16 ± 0.57	3.35 ± 0.73	3.46 ± 1.05
Salivary Glands	2.27 ± 0.31	1.43 ± 0.27	1.19 ± 0.11
**Organ**	**1 h**	**3 h**	**6 h**
Blood	3.87 ± 0.55	2.18 ± 0.20	1.87 ± 0.17
Small Bowel	2.34 ± 0.19	1.67 ± 0.45	1.21 ± 0.03
Large Bowel	0.66 ± 0.07	0.96 ± 0.45	1.05 ± 0.23
Kidneys	32.5 ± 1.78	32.06 ± 6.97	30.39 ± 5.82
Spleen	0.64 ± 0.12	0.41 ± 0.41	0.57 ± 0.10
Stomach	0.50 ± 0.10	0.41 ± 0.16	0.39 ± 0.03
Liver	11 ± 0.73	8.22 ± 2.92	8.54 ± 1.35
Muscle	0.45 ± 0.05	0.24 ± 0.11	0.22 ± 0.07
Bone	0.21 ± 0.01	0.12 ± 0.04	0.13 ± 0.06
Lungs	0.67 ± 0.05	0.38 ± 0.09	0.39 ± 0.15
Salivary Glands	0.27 ± 0.08	0.14 ± 0.01	0.12 ± 0.01

**Table 4:** *p*-values resulting from multiple statistical comparison of T/B ratios obtained in vivo in mice infected with different amounts of Gram- or Gram+ bacteria (10^7^, 10^8^ and 10^9^) and analyzed at different time-points (1, 3 and 6 h) after i.v. injection of with ^99m^Tc-HYNIC-PMB.

	**1 h**					
**Bacterial Strain**	***E. faecalis***	***S. aureus***	***E. faecalis***	***S. aureus***	***E. faecalis***	***S. aureus***
	**10^7^**		**10^8^**		**10^9^**	
*A. baumanii*	ns	ns	ns	ns	ns	ns
*P. aeruginosa*	ns	ns	<0.0001	0.04	ns	ns
*E. coli*	ns	ns	0.0008	ns	ns	ns
			**3 h**			
**Bacterial Strain**	***E. faecalis***	***S. aureus***	***E. faecalis***	***S. aureus***	***E. faecalis***	***S. aureus***
	**10^7^**		**10^8^**		**10^9^**	
*A. baumanii*	ns	ns	ns	ns	ns	ns
*P. aeruginosa*	ns	ns	0.0002	0.0007	0.02	0.006
*E. coli*	ns	ns	ns	ns	ns	ns
			**6 h**			
**Bacterial Strain**	***E. faecalis***	***S. aureus***	***E. faecalis***	***S. aureus***	***E. faecalis***	***S. aureus***
	**10^7^**		**10^8^**		**10^9^**	
*A. baumanii*	ns	ns	0.007	0.007	0.01	0.04
*P. aeruginosa*	ns	ns	0.01	0.027	0.001	0.001
*E. coli*	ns	ns	0.004	0.004	0.002	0.003

Multiple comparison was performed between different T/B, as shown in Figure 7. Gram-negative (*A. baumanii, P. aeruginosa* and *E. coli*) vs. Gram-positive (*E. faecalis* and *S. aureus*).

## Data Availability

All data are available from our statistician G.C.
